# Modification of Polylactide with Triblock and Diblock Copolymers of Ethylene Glycol and Propylene Glycol

**DOI:** 10.3390/ijms262110422

**Published:** 2025-10-27

**Authors:** Miroslaw Pluta, Joanna Bojda, Ewa Piorkowska

**Affiliations:** Centre of Molecular and Macromolecular Studies, Polish Academy of Sciences, Sienkiewicza 112, 90-363 Lodz, Poland; joanna.bojda@cbmm.lodz.pl

**Keywords:** polylactide, plasticization, block copolymers, PPG-b-PEG-b-PPG, PPG-b-PEG

## Abstract

Polylactide (PLA) was melt blended with block copolymers of ethylene glycol and propylene glycol: a triblock copolymer (PPG-b-PEG-b-PPG) with a molar mass of 2700 g/mol and 40 wt% PEG content, and a diblock copolymer (PPG-b-PEG) with a molar mass of 4000 g/mol and 50 wt% PEG content. The structure as well as the thermal and mechanical properties of both amorphous and crystallized blends were investigated. Due to the copolymers’ chemical composition and the resulting phase structure, the 10 wt% amorphous blends with PPG-b-PEG-b-PPG and PPG-b-PEG, with T_g_ values of 38 °C and 46 °C, respectively, exhibited relatively high yield stress, close to 45 MPa, along with remarkable elongation at break. Notably, the blend with the triblock copolymer showed a 70-fold increase in elongation at break compared to neat amorphous PLA. Furthermore, the tensile impact strength of the blend with the diblock copolymer surpassed that of neat PLA. Upon crystallization, the 10 wt% blends showed reduced yield stress and elongation at break; however, the elongation at break exceeded 7–25 times that of neat crystalline PLA. Furthermore, their tensile impact strength increased to more than three times the value of crystalline PLA.

## 1. Introduction

The development of biobased polymers has been a subject of intensive research, e.g., [1,2,3,4,5,6], due to their role in reducing carbon dioxide emissions, which contribute to global warming, as well as in decreasing dependence on fossil resources. Among them, polylactides (PLAs) have attracted significant attention because they can be produced from annually renewable natural resources [7] and are biodegradable, compostable, and recyclable [8,9,10,11]. Owing to these advantages, PLAs became an eco-friendly alternative to petroleum-based polymers. However, their application in areas requiring enhanced ductility and toughness is limited by stiffness and brittleness due to the relatively high glass transition temperature (T_g_), typically in the range of 55 ÷ 60 °C [12].

To overcome this drawback, several approaches have been employed: copolymerization, e.g., [13,14,15,16], blending with immiscible polymers (including reactive blending), e.g., [12,17,18,19,20,21,22,23,24], and plasticization by incorporating various low- and high-molar-mass plasticizers, e.g., [17,25,26,27,28,29,30,31,32,33,34,35,36,37,38,39,40,41]. While these methods improve the ductility and toughness of PLA, they generally lead to a reduction in the elastic modulus and tensile yield stress compared to neat PLA homopolymer. Blending PLA with immiscible or partly miscible polymers results in phase-separated “rubber-toughened” blends, where the dispersed minor phase initiates localized plastic deformation of the glassy PLA matrix under load in the form of crazes or shear bands. In such systems, the decrease in T_g_ of the continuous phase is usually minor but the presence of the dispersed phase often reduces transparency. By contrast, efficient plasticization enhances the segmental mobility of polymer chains, leading to a more pronounced decrease in T_g_. This improves PLA’s flexibility and ductility but also results in a stronger reduction in the yield stress and elastic modulus compared to rubber-toughened blends. Numerous plasticizers with diverse chemical structures and molecular masses have been reported, including glycerol [27], citrate esters [28], triacetine and tributyl citrate [29], poly(ethylene oxide) [30], poly(ethylene glycol) (PEG) [26,27,31,32,33,34], poly(propylene glycol) (PPG) [35,36], cardanol [37], plant oils [38,39,40], and epoxidized isobutyl esters [41].

One of the drawbacks of plasticized PLAs is aging, which alters their physical properties. This phenomenon is often associated with plasticizer migration, phase separation, or the crystallization of plasticizers and PLA [33,42,43,44]. To mitigate migration and leaching, plasticizers with higher molar mass and/or more complex structure have been employed, though these can promote or intensify phase separation. Furthermore, a lowered T_g_ narrows the application temperature window of PLA. This limitation can be overcome by the crystallization of plasticized PLA, since the upper temperature limit of crystalline PLA applications is determined by the melting of PLA crystals.

Optically pure poly(l-lactide) (PLLA) and poly(d-lactide) are crystallizable polymers; however, a decrease in optical purity worsens the crystallizability of PLAs. Slowly crystallizing PLAs can be quenched to the glassy state without undergoing crystallization and subsequently cold crystallized upon heating. Usually, the cold crystallization temperature of plasticized PLA, which reflects its ability to crystallize, decreases in parallel with T_g_. In general, although the crystallization of PLA in a plasticizer-containing blend can broaden its application temperature range, it is often accompanied by the redistribution of the plasticizer [34,36]. This redistribution can lead to the accumulation of the plasticizer between PLA spherulites, weakening the material and negatively affecting its ultimate mechanical properties. Excessive local accumulation of the plasticizer can also cause phase separation, which can be detrimental to drawability and toughness, particularly when it is accompanied by the crystallization of the plasticizer. Consequently, crystalline PLAs with a plasticized amorphous phase often exhibit relatively low elongation at break, especially when compared to their amorphous counterparts.

Plasticization of PLA with hydrophilic PEG [26,27,31,32,33,34] has been extensively investigated due to its non-volatility, possible biobased origin, good miscibility with PLA, and biodegradability [45,46]. However, if the phase separation and crystallization of PEG occur in PLA/PEG blends, their mechanical performance deteriorates [42,43]. In contrast, hydrophobic PPG, also biodegradable [47,48] and possibly biobased, is unable to crystallize because of atacticity, but its miscibility with PLA strongly decreases with increasing molar mass [35]. Previous studies have shown that the random and block copolymers PEG-b-PPG-b-PEG with varying ratios of hydrophilic (PEG) and hydrophobic (PPG) blocks efficiently enhance the drawability and tensile impact strength of PLA [49,50,51,52]. The miscibility of these copolymers with PLA depends on their molar mass and PEG content. Interestingly, the best drawability was achieved for partially miscible blends, in which fine liquid inclusions of the modifier were dispersed in a PLA rich matrix, in which T_g_ decreased only moderately to about 45–50 °C. At a relatively low modifier content (10 wt%), a 37-fold increase in elongation at break and a 1.5-fold increase in tensile impact strength compared to neat PLA were observed [51]. Both the molecularly dispersed fractions of the copolymers, which plasticized the PLA matrix, and the phase-separated fractions forming fine liquid inclusions contributed to the enhanced plastic deformation of the blends. Crystallization, however, reduced the drawability and toughness of the blends. Nevertheless, even the crystalline blends exhibited good drawability, with the elongation at break and tensile impact strength exceeding those of neat crystalline PLA [52].

In the present study, we investigated the modification of PLA with block copolymers of ethylene glycol and propylene glycol, which differ in chemical structures and possess a higher PEG content than those used previously [50,51,52]. Two copolymers were employed: a triblock copolymer PPG-b-PEG-b-PPG, with a molar mass of 2700 g/mol and a PEG content of 40 wt%, and a diblock copolymer PPG-b-PEG, with a molar mass of 4000 g/mol and a PEG content of 50 wt%. Both the PEG content and the copolymer structure were expected to influence the miscibility with PLA and, consequently, the properties of the modified PLA.

Both amorphous and crystalline blends were prepared, and their structure, thermal, and mechanical properties were examined. Due to the chemical structure of the copolymers and the resulting phase morphology of the blends, remarkable drawability was achieved while maintaining a relatively high yield stress. Notably, the crystallized blends exhibited not only elongation at break values significantly higher than that of neat crystalline PLA but also a tensile impact strength more than three times greater than that of crystalline PLA.

## 2. Results and Discussion

### 2.1. Thermal Properties

**Figure 1:**
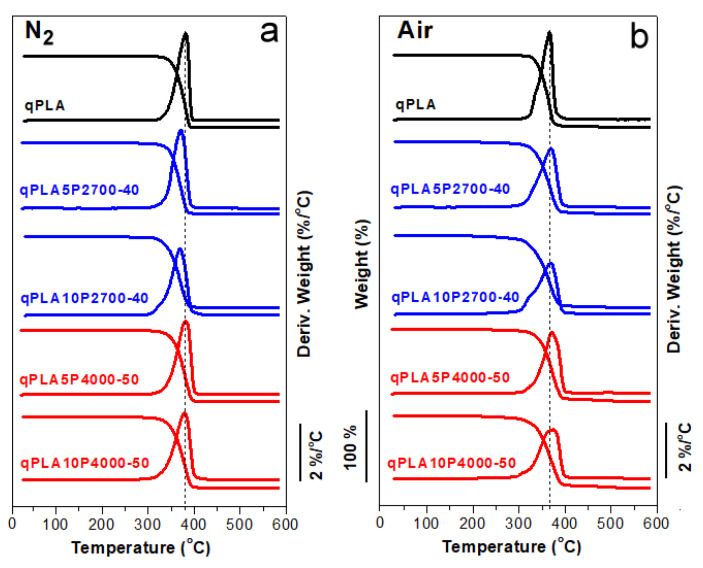
TGA and DTGA thermograms of PLA and its blends with P2700-40 and P4000-50 copolymers in nitrogen (**a**) and air (**b**), respectively.

The TGA and DTGA thermograms of qPLA and its blends with the copolymers recorded in an inert nitrogen atmosphere and in air are shown in Figure 1a,b, while the temperatures of 5% weight loss (T_5%_) and the DTGA peak temperatures (T_d_) are listed in Table 1. In nitrogen, the T_5%_ and T_d_ values of neat qPLA and the blends ranged from 322 to 340 °C and from 369 to 382 °C, respectively. In air, the values recorded were somewhat lower, ranging from 295 to 331 °C for T_5%_ and from 367 to 375 °C for T_d_. Nevertheless, all materials exhibited thermal stability within temperature ranges, in which they were melt blended and compression molded.

**Figure 2:**
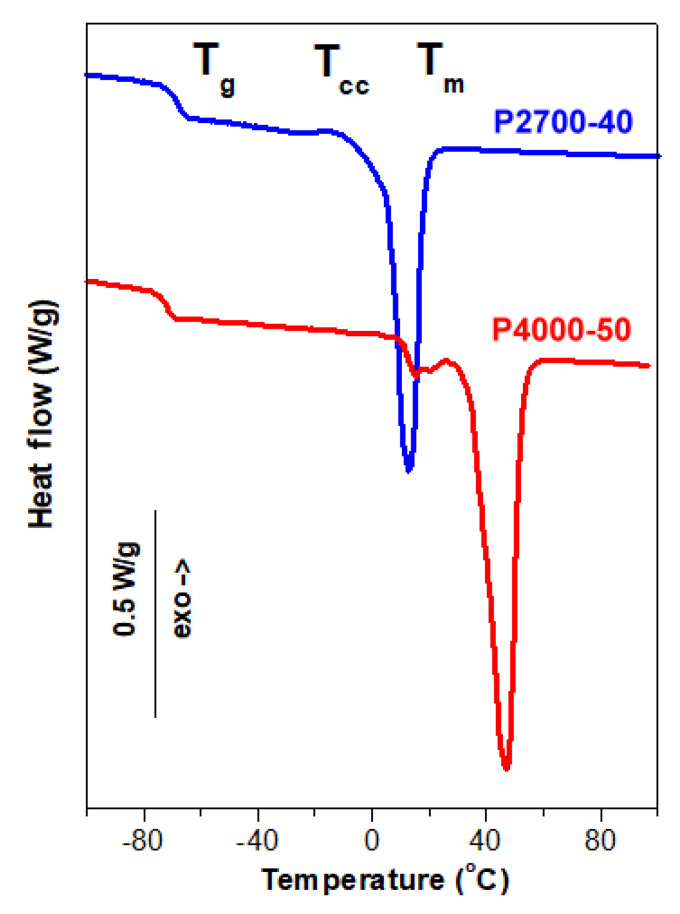
DSC heating thermograms of P2700-40 and P4000-50 copolymers recorded at 10 °C/min.

**Figure 3:**
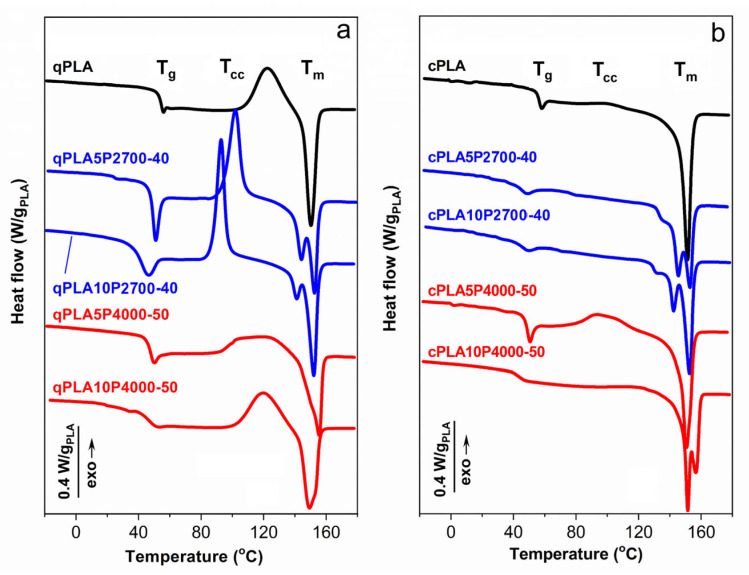
DSC heating thermograms of PLA and its blends with P2700-40 and P4000-50 copolymers, quenched (**a**) and crystallized (**b**), recorded at 10 °C/min. Thermograms vertically shifted for clarity.

The DSC thermograms of the materials studied are presented in Figure 2 and Figure 3, while the corresponding calorimetric data are collected in Table 2 and Table 3. The degree of crystallinity (X_cPLA_) of PLA in the materials was calculated assuming the enthalpy of fusion of PLA crystals of 106 J/g [53]. 

The heating thermograms of the copolymers P2700-40 and P4000-50, shown in Figure 2, demonstrated their different thermal behaviors. The T_g_ of the P4000-50 copolymer at −72 °C was lower than that of P2700-40, at −68 °C. The thermogram of the P2700-40 copolymer exhibited a small exotherm, likely due to cold crystallization, at around −13 °C with a low enthalpy of 1.5 J/g. This exotherm was followed by a melting endotherm with a peak temperature (T_m_) of 13 °C and a melting enthalpy (ΔH_m_) of 43 J/g. In contrast, the thermogram of the P4000-50 copolymer showed only two melting peaks, a small one with a T_m_ of 26 °C and the main one with a T_m_ of 47 °C with a total ΔH_m_ of 74 J/g. It should be emphasized that, in these copolymers, only PEG blocks are capable of crystallization. Therefore, the crystallinity of the copolymers (X_c_) and the crystallinity of the PEG blocks (X_cPEG_) were calculated assuming the enthalpy of fusion of the PEG crystals of 196.8 J/g [54]. The crystallization and melting of the PEG blocks were strongly influenced by the copolymer structure. The X_cPEG_ and T_m_ of the PEG central blocks in the triblock copolymer, with an average molar mass of approx. 1100 g/mol, were lower than those of the longer PEG blocks in the diblock copolymer, with an average molar mass of 2000 g/mol.

The DSC heating thermograms of the quenched PLA and its blends in Figure 3a,b show the glass transitions, cold crystallization exotherms, and melting peaks. The cold crystallization enthalpy (ΔH_cc_) was nearly equal to ΔH_m_, indicating that the PLA in these materials was amorphous. The T_g_ of qPLA was 53 °C, while in the 5 wt% blends it decreased to approx. 46–48 °C. Increasing the P2700-40 content in the blend to 10 wt% caused a further decrease in T_g_ to 38 °C. The reduction in T_g_ confirms the plasticization of the PLA in the blends with the copolymers. In contrast, the T_g_ of qPLA10P4000-50, at 46 °C, remained the same as of the corresponding 5 wt% blend. This suggests that the copolymer content in the PLA-rich phase did not increase with copolymer loading, indicating phase separation.

The qPLA cold crystallization exotherm with the peak temperature (T_cc_) at 122 °C was followed by a melting peak with T_m_ at 150 °C. Plasticization with the P2700-40 copolymer shifted the cold crystallization of the quenched blends, to lower temperatures. The blends exhibited a T_cc_ at 102 °C and 93 °C, decreasing with increasing copolymer content, both below that of qPLA. Cold crystallization began at lower temperatures and the peaks were sharper, indicating faster crystallization than in qPLA, as previously reported for plasticized PLA [26,51,54]. This resulted in a higher crystallinity of PLA in the blends (X_cPLA_), reflected in ΔH_cc_, of approx. 30 J/g_PLA_, exceeding that of qPLA, of approx. 26 J/g. The lower crystallization temperature of these blends led to double melting peaks, with T_m_ near 140 °C and 150 °C. Such melting behavior of PLA was explained by others [55] as a result of the reorganization within the orthorhombic α-phase of PLA, namely, melting and re-crystallization processes occurring during the heating scan [55,56,57]. In contrast, the cold crystallization exotherms of blends with the P4000-50 copolymer were low and broad, centered at approx. 120 °C and 113 °C, and ΔH_cc_ of approx. 21 J/g_PLA_ and 29 J/g_PLA_, respectively. These broad exotherms indicated that the cold crystallization occurred over a wide temperature range, extending up to the melting peaks with T_m_ around 155 °C. Such behavior may be related to the relatively high T_g_ of the PLA-rich phase and phase separation, since the presence of inclusions of the second polymer can disturb spherulite growth [58,59].

The thermograms of the crystallized materials, shown in Figure 3b, exhibited glass transitions and melting peaks, except for the neat cPLA and the cPLA5P4000-50 blend, for which small cold crystallization exotherms were observed, indicating that crystallization was not entirely completed during thermal treatment. Upon crystallization, the T_g_ of neat PLA increased to 55 °C. Crystallization led to an increased copolymer content in the amorphous phase, which should result in a decrease in T_g_. However, a decrease was observed only for the cPLA5P2700-40 blend, while, in other cases, T_g_ either remained unchanged or even increased. This suggests that the copolymer content in the PLA-rich phase did not increase, or even decreased due to enhanced or developed phase separation, as further confirmed by the DMTA and SEM analyses. The thermograms exhibited main melting peaks, with T_m_ above 150 °C. Except for the cPLA5P4000-50 blend, additional melting peaks were observed, with T_m_ at 142–145 °C or 156 °C. For the blends with the P2700-40 copolymer, these peaks were accompanied by low-temperature shoulders, most likely related to the melting of thin crystals formed during post-crystallization cooling. The ΔH_m_ of all crystallized materials ranged from 34 to 36 J/g_PLA_. The crystallinity level of PLA in the materials, calculated based on ΔH_m_ or ΔH_m_ − ΔH_cc_, where applicable, was 31–34%, except for the cPLA5P4000-50 blend, for which it was about 23%.

### 2.2. Dynamic Mechanical Thermal Properties

**Figure 4:**
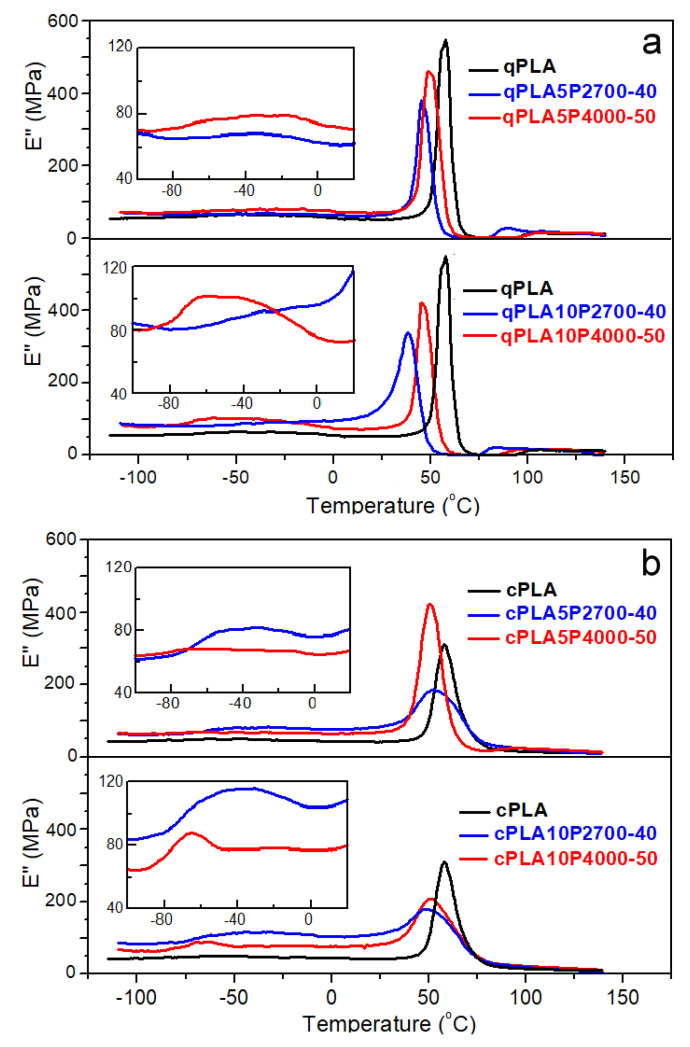
Temperature dependencies of loss modulus, E″, of PLA and its blends with P2700-40 and P4000-50 copolymers, quenched (**a**) and crystallized (**b**) at 1 Hz and 2 °C/min.

The temperature dependencies of the loss modulus (E″) of the neat PLA and its blends with the copolymers are shown in Figure 4a,b, while the temperatures (T_HE″_ and T_LE″_) of the high- and low-temperature peaks of these curves are listed in Table 3.

The E″ plots of all quenched materials exhibited peaks in the glass transition region. For qPLA, a single peak was observed, with T_HE″_ at 57 °C, whereas the peaks of the blends appeared at lower temperatures. The addition of 5 wt% of P2700-40 and P4000-50 copolymers shifted the peaks to lower temperatures, and T_HE″_ to 45 °C and 49 °C, respectively. Moreover, in the latter case, an additional low and broad low-temperature peak appeared, with a maximum at T_LE″_ of −32 °C. Increasing the P2700-40 and P4000-50 content to 10 wt% caused the high-temperature peaks to shift further towards lower temperatures, decreasing their T_HE″_ to 39 °C and 46 °C, respectively. In addition, a low-temperature shoulder developed on the qPLA10P2700-40 peak, whereas, for qPLA10P4000-50, the low-temperature peak intensified and its T_LE″_ shifted to −50 °C. The appearance of the low-temperature shoulder and additional low-temperature peaks indicates the presence of the copolymer-rich phase resulting from phase separation, particularly in the blends with the P4000-50 copolymer. The main high-temperature peaks of the E″ plots of crystallized materials were lower and broader than those of their amorphous counterparts, which can be attributed to the reduced amount of amorphous phase and the broadening of the glass transition. This difference was smallest for the cPLA5P4000-50 blend due to its relatively low crystallinity. T_HE’_ decreased from 58 °C for cPLA to 51–52 °C for 5 wt% blends, and to 49–51 °C for 10 wt% blends. In addition, low-temperature peaks with maxima close to −40 °C appeared on the curves of blends with the P2700-40 copolymer, indicating that crystallization enhanced phase separation by increasing the copolymer content in the amorphous phase. The enhancement of phase separation in the cPLA10P4000-50 blend led to the narrowing and shifting of the low-temperature peak towards lower temperatures. Its T_LE″_ of −64 °C was closer to the T_g_ of the copolymer than in the case of the other blends.

### 2.3. Morphology

**Figure 5:**
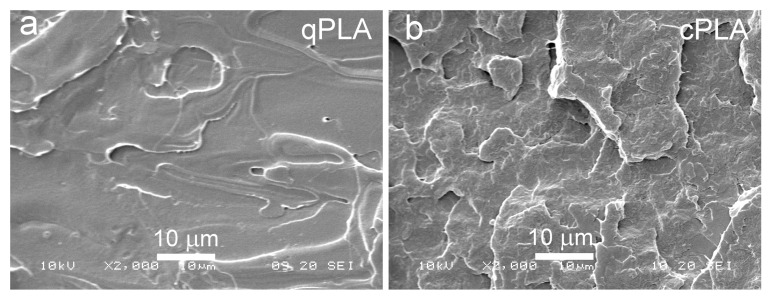
SEM micrographs of cryo-fractured surfaces of qPLA (**a**) and cPLA (**b**).

**Figure 6:**
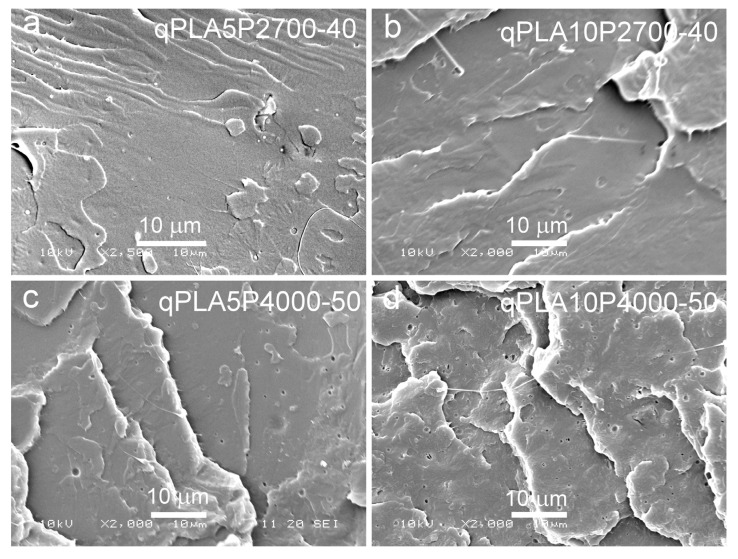
SEM micrographs of cryo-fracture surfaces of quenched blends of PLA with P2700-40 and P4000-50 copolymers: qPLA5P2700-40 (**a**), qPLA10P2700-40 (**b**), qPLA5P4000-50 (**c**), and qPLA10P4000-50 (**d**).

**Figure 7:**
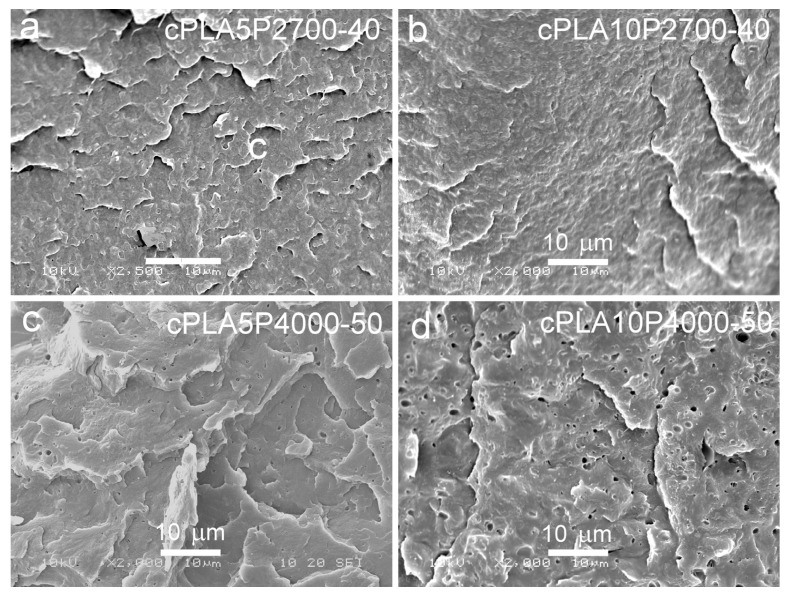
SEM micrographs of cryo-fracture surfaces of crystallized blends of PLA with P2700-40 and P4000-50 copolymers: cPLA5P2700-40 (**a**), cPLA10P2700-40 (**b**), cPLA5P4000-50 (**c**), and cPLA10P4000-50 (**d**).

SEM micrographs of cryo-fractured materials are shown in Figure 5, Figure 6 and Figure 7. The cryo-fracture surface of the qPLA and the quenched blends are relatively smooth, whereas those of their crystalline counterparts were more developed due to the presence of crystalline aggregates. The micrographs of quenched blends with the P2700-40 copolymer revealed a homogeneous structure without visible inclusions. This observation is in accordance with the E″ plots shown in Figure 4, which exhibit single peaks, with T_HE″_ decreasing as the copolymer content increased, indicating plasticization. This effect was also corroborated by the T_g_ values determined from the DSC thermograms. The low-temperature shoulder observed in the E″ plot of the qPLA10P2700-40 blend indicated the presence of the copolymer-enriched phase, but no distinct domains were detected by SEM. In contrast, the qPLA5P4000-50 and qPLA10P4000-50 blends exhibited small holes up to 1 μm and 1.5 μm in size, respectively, where the copolymer-rich phase accumulated. The E″ plots of these blends showed low and broad low-temperature peaks, suggesting a wide distribution of relaxation times, most likely related to compositional gradients within the copolymer-rich phase. SEM analysis confirmed the presence of a minor separated phase, probably corresponding to the richest copolymer regions, forming discrete inclusions. The same applies to the cPLA5P2700-40, cPLA10P2700-40, and cPLA5P4000-50 blends. In the blends containing the P2700-40 copolymer, a few holes with sizes below 0.5 μm were detected at higher magnifications, while, in the cPLA5P4000-50 blend, the holes were more numerous and larger, up to 1 μm. In the cPLA10P4000-50 blend, such holes were much more numerous and reached sizes of up to 2 μm. This finding is consistent with the pronounced low-temperature E″ peak observed for this material, with the T_LE″_ close to the T_g_ of the copolymer determined by DSC.

The obtained result indicates that the miscibility of the diblock P4000-50 copolymer with PLA was lower than that of triblock the P2700-40 copolymer, despite its higher PEG content. This effect can be attributed to the higher molar mass and longer PPG blocks, which reduced the miscibility. Furthermore, crystallization increased the copolymer content in the amorphous phase and intensified phase separation in the blends, particularly in those containing the P4000-50 copolymer.

### 2.4. Tensile and Tensile Impact Properties

**Figure 8:**
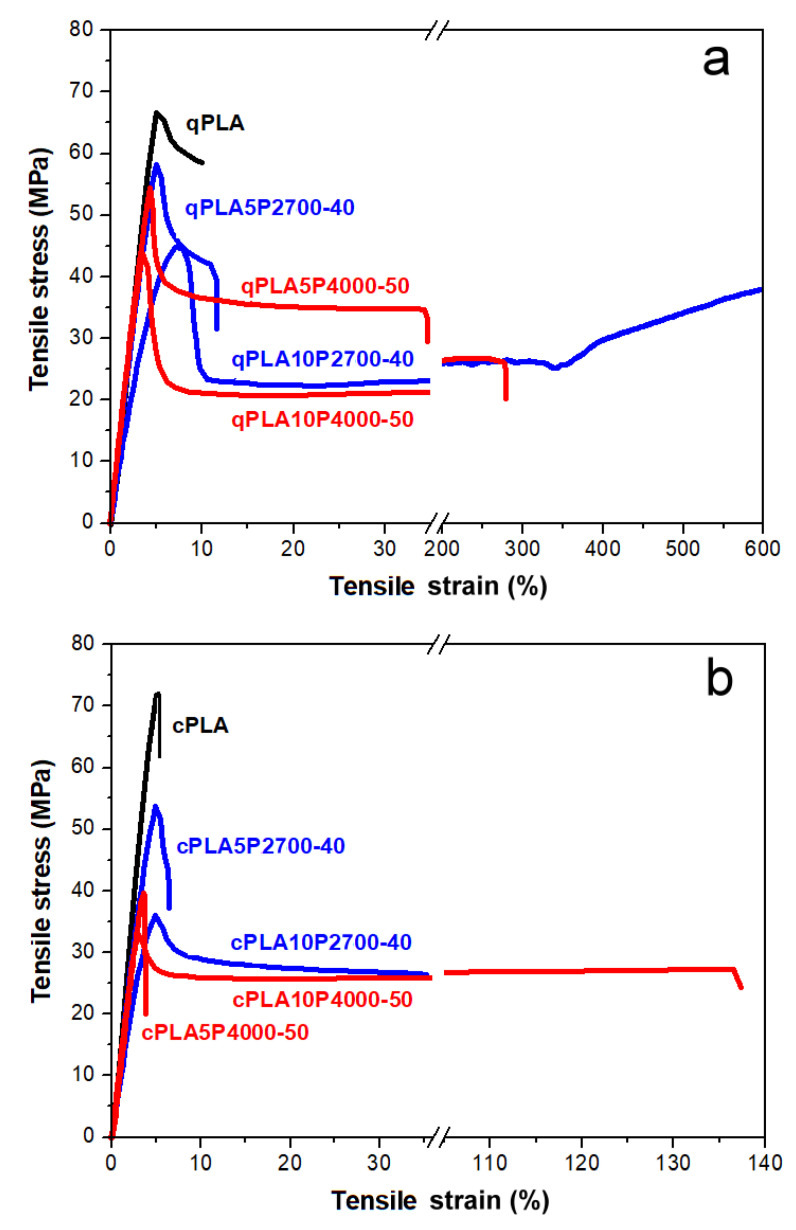
Stress–strain dependencies of neat PLA and its blends with P2700-40 and P4000-50 copolymers: quenched (**a**) and crystallized (**b**).

The exemplary engineering stress–engineering strain dependencies of quenched and crystallized PLA and its blends with the P2700-40 and P4000-50 copolymers are shown in Figure 8a,b, while the average mechanical parameters—including the elastic modulus (E), yield strain and stress (ε_y_, σ_y_), strain and stress at break (ε_b_, σ_b_), and tensile impact strength (U)—are collected in Table 4.

The stress–strain dependencies of all materials, except for cPLA and the cPLA5P4000-50 blend, exhibited a pronounced yield, beyond which the stress dropped. Therefore, their tensile strength was determined by σ_y_. Differences in the mechanical properties of the tested materials become apparent already at the initial stage of deformation. The qPLA exhibited an E of 1.45 GPa and only a weak ability for plastic deformation, with a σ_y_ of 67 MPa, and ε_b_ and σ_b_ of 8% and 60 MPa, respectively. Crystallinity slightly increased E to 1.55 GPa, but the early fracture of cPLA occurred before yielding, at an ε_b_ and σ_b_ of 5% and 68 MPa, respectively. In addition, U decreased significantly from 65 to 34 kJ/m^2^. All quenched blends exhibited a lower E, 0.87–1.35 GPa, and an improved ability for plastic deformation compared to qPLA. This improved ability for plastic deformation was reflected in the decreased σ_y_, 44–59 MPa, and in the increased ε_b_. While the ε_b_ of the qPLA5P2700-40 and qPLA5P4000-50 blends was only 11% and 35%, respectively, increasing the copolymer content to 10 wt% dramatically improved the ε_b_ to 560% and 240%, respectively. The σ_b_ values of all quenched blends were lower than that of qPLA, 26–44 MPa, and decreased with an increasing copolymer content. During the drawing of the qPLA10P2700-40 blend, strain-hardening occurred above 300% strain, resulting in σ_b_ of 38 MPa, which was higher than in the case the qPLA10P4000-50 blend, 26 MPa. In addition, the U of the quenched blends was improved, especially for the blends with the P4000-50 copolymer, to 90–95 kJ/m^2^.

Upon crystallization, the E of the blends changed only by a few percent, which can be attributed to the crystallinity and to the crystallization-driven increase in the copolymer content in the amorphous phase. The latter effect reduced the σ_y_, especially in the case of the 10 wt% blends, for which σ_y_ decreased to 34–35 MPa. However, the crystallized 5 wt% blends fractured early (the cPLA5P4000-50 blend fractured even before yielding) at ε_b_ values of only few percent and σ_b_ values far below that of cPLA. Those ε_b_ values were significantly lower, whereas the σ_b_ values were only slightly higher—by 2–3 MPa—compared to the quenched 5 wt% blends. Increasing the copolymer content to 10 wt% enlarged ε_b_ to 36% for the cPLA10P2700-40 blend and to 123% for the cPLA10P4000-50 blend, while σ_b_ decreased to 26–27 MPa. Although these ε_b_ values were lower than those of the quenched 10 wt% blends, they still exceeded that of cPLA by approx. 7 and 25 times, respectively. Upon crystallization, the U of the 5 wt% blends decreased to 55–57 kJ/m^2^, but in the case of the 10 wt% blends, increased to 107–120 kJ/m^2^. Nevertheless, the U of each crystallized blend exceeded that of cPLA.

The obtained results indicate that, in the case of the quenched blends, an enhancement in the drawability was already achieved at a 5 wt% of copolymer content. However, increasing the copolymer content to 10 wt% led to a significant improvement, especially for the qPLA10P2700-40 blend, whose ε_b_ reached 560%—a higher value than that reported for PLA modified with the same amount of PEG-b-PPG-b-PEG copolymers [51]. In addition, the decrease in σ_y_ compared to qPLA was moderate, to 44–46 MPa. Among the quenched blends, the T_g_ of the qPLA10P2700-40 blend, at 38 °C, was the lowest, indicating the most efficient plasticization. In turn, the qPLA10P4000-50 blend exhibited not only good drawability but also an improved U of 90 kJ/m^2^. Upon crystallization, the ε_b_ of all the blends worsened, despite the crystallization-driven increase in the copolymer content in the amorphous phase and a decrease in σ_y_. Nevertheless, the ε_b_ values of the 10 wt% blends exceeded that of cPLA. Moreover, crystallization increased the U values of these blends to more than three times that of cPLA. Particularly, the cPLA10P4000-50 blend exhibited both high ε_b_ and U, at 123% and 107 kJ/m^2^, respectively. Such properties were not achieved for crystalline blends with PEG-b-PPG-b-PEG copolymers [50,52].

Phase separation in the cPLA10P2700-40 blend and in both 10 wt% blends with the P4000-50 copolymer was evidenced by DMTA and SEM. The presence of a copolymer-rich phase with a low T_g_, dispersed in the amorphous PLA-rich phase of both quenched and crystallized blends—despite the relatively high T_g_ value of 46 °C—proved beneficial for the mechanical performance. Under ambient conditions, the P4000-50 copolymer is semicrystalline, with 38% crystallinity, and exhibits a thick-paste consistency, whereas the P2700-40 copolymer is a thick liquid. Soft inclusions are known to toughen glassy amorphous polymers. In the studied blends, both the molecularly dispersed fractions of the copolymers, which plasticized the amorphous phase, and the phase-separated fractions of the copolymers, which acted as soft inclusions, contributed to the enhanced ability for plastic deformation of these partially miscible and phase-separated blends, which enabled improved drawability and toughness.

## 3. Materials and Methods

### 3.1. Materials

The polylactide (PLA) used in this study was the commercially available grade 2002D from NatureWorks LLC (Minnetonka, MN, USA). According to the manufacturer [60], it has a density of 1.24 g/cm^3^ and a melt flow index of 5–7 g/10 min (210 °C, 2.16 kg, ASTM D1238 standard [61]). Its weight average molar mass (M_w_) and dispersity (M_w_/M_n_) were 104 kg/mol and 1.4, respectively, as determined by size-exclusion chromatography (SEC) with a multi-angle laser light scattering (MALLS) detector in dichloromethane. The optical rotation measurements indicated a d-lactide content of 2.5% and an l-lactide content of 97.5%.

PLA was modified with two copolymers of different block structures: Pluronic^®^ 17R4 and Pluriol^®^ WSB125, both supplied by BASF (Ludwigshafen, Germany). Pluronic^®^ 17R4 is a triblock copolymer composed of two poly(propylene glycol) (PPG) end blocks and a central poly(ethylene glycol) (PEG) block (PPG-b-PEG-b-PPG). Due to the presence of both hydrophilic (PEG) and hydrophobic (PPG) segments, it exhibits amphiphilic behavior. Pluronics are commonly employed as surfactants in the pharmaceutical industry. Pluriol^®^ WSB125 is a diblock copolymer consisting of PPG and PEG blocks (PPG-b-PEG). It is typically used in formulations such as emulsifier concentrates, emulsions (oil-in-water), microemulsions, suspension concentrates, soluble liquids, and wettable powders. The main characteristics of the copolymers, including their nominal molar mass, PEG content, and assigned codes (P2700-40 for Pluronic^®^17R4 and P4000-50 for Pluriol^®^ WSB125), are summarized in Table 5. The code notation corresponds to molar mass and PEG content, respectively. Analysis of Pluronic^®^ 17R4 with SEC yielded a number average molar mass (M_n_) of 2700 g/mol and dispersity of 1.06 [62].

### 3.2. Blend and Sample Preparation

PLA and the copolymers were dried under reduced pressure at 100 °C for 4 h. Subsequently, the blends of PLA with the copolymers were prepared using a Brabender mixer (Duisburg, Germany) operated at 190 °C for 15 min at 60 rpm in an inert nitrogen atmosphere to reduce degradation. The copolymer contents in the blends, 5 and 10 wt%, were selected based on preliminary experiments. Neat PLA was also processed under the same conditions as a reference material.

The blends are denoted as, for example, PLA5P2700-40, where the first number indicates the copolymer content and the code refers to the copolymer.

For further testing, films with thicknesses of 0.5 mm and 1 mm were compression molded at 180 °C for 3 min in a hydraulic press and then rapidly quenched between metal blocks kept at room temperature. This procedure allowed us to obtain amorphous films marked with the letter q, for example, qPLA (quenched PLA). Some of quenched films were subsequently heated between metal blocks at a rate of approx. 8 °C/min to 120 °C, held at this temperature for 2–6 min, and quenched to room temperature. These materials, which cold crystallized during this thermal treatment, were denoted with the letter c as, for example, cPLA (crystallized PLA). Cold crystallization was chosen as a crystallization method because it promotes more intense spherulite nucleation, resulting in a shorter crystallization time and smaller spherulites [63].

### 3.3. Characterization

Thermogravimetric analysis (TGA) was carried out for all materials with a TGA Q50 (TA Instruments, New Castle, DE, USA). Samples were heated from room temperature up to 600 °C at a rate of 20 °C/min, both in air and under nitrogen.

Thermal properties of the copolymers, neat PLA, and their blends were analyzed using differential scanning calorimetry (DSC 2920, TA Instruments, New Castle, DE, USA). Measurements were performed at a heating rate of 10 °C/min in the range from –50 to 190 °C under nitrogen flow. T_g_ values were determined by the half-height method.

Dynamic mechanical thermal analysis (DMTA) was conducted on rectangular specimens (28 mm × 10 mm) cut from 1 mm thick films. An Mk III DMTA apparatus (Rheometric Scientific, Epsom, UK) was used in dual cantilever bending mode at a frequency of 1 Hz during heating from −100 to 140 °C at 2 °C/min.

Analysis of morphology was performed on cryo-fractured 1 mm thick films. The exposed surfaces were sputter-coated with gold and observed using a scanning electron microscope (SEM) 5500LV (JEOL, Tokyo, Japan).

Tensile measurements were performed using an Instron 5582 testing machine (Instron Corp., High Wycombe, UK) at room temperature and a crosshead speed of 50%/min. At least five specimens of each material were tested. Oar-shaped specimens conforming to ISO 527 type 1BA [64], with a 25 mm gauge length, were cut from 0.5 mm thick films. The values of elastic modulus were determined from the linear region of the stress–strain curves.

Tensile impact tests were conducted at room temperature on 8–10 specimens of each material using a Resil 5.5 impact tester (CEAST, Charlotte, NC, USA), equipped with a hammer of maximum energy of 1 J and velocity of 2.9 m/s. Specimens, cut from 0.5 mm thick films, conformed to ISO 8256 [65] with the following dimensions: total length of 80 mm, narrow section length of 30 mm, and width of 10 mm.

## 4. Conclusions

PLA was efficiently modified by melt blending with 5 wt% and 10 wt% of commercially available block copolymers of ethylene glycol and propylene glycol: PPG-b-PEG-b-PPG (P2700-40) and PPG-b-PEG (P4000-50), with molar masses of 2700 g/mol and 4000 g/mol, respectively, and P_c_ values of 40 wt% and 50 wt%, respectively. The blends were thermally stable, with T_5%_ values close to or above 300 °C, both in air and nitrogen atmosphere. By quenching the melt, amorphous PLA blends were prepared, while cold crystallization resulted in blends with crystalline PLA. The triblock copolymer was better miscible with PLA than the diblock one. No distinct inclusions were found in quenched blends with the triblock copolymer, and only a low-temperature shoulder of the E’’ peak was observed for the 10 wt% blend. In contrast, blends with the diblock copolymer exhibited inclusions of a separated phase and low-temperature E″ peaks, evidencing phase separation. Upon crystallization, phase separation occurred or was enhanced, resulting in the presence of phase-separated inclusions and low-temperature E″ peaks. In the cPLA10P4000-50 blend, this peak was relatively narrow, with the T_LE″_ close to the T_g_ of the copolymer, and the inclusions were the largest—though still below 2 μm in size.

At a 10 wt% copolymer content, a dramatic improvement in the drawability of the quenched blends was observed, especially for the qPLA10P2700-40 blend, which exhibited a ε_b_ of 560%, while the decrease in σ_y_ was moderate, to 44–46 MPa. The lowest T_g_ of this blend, at 38 °C, indicated the most efficient plasticization. However, the qPLA10P4000-50 blend exhibited not only good drawability but also an improved U of 90 kJ/m^2^. Upon crystallization, the drawability of all the blends worsened and the ε_b_ decreased; however, in the case of the 10 wt% blends, it still exceeded that of cPLA by several times. The U of these blends increased to values more than three times higher than that of cPLA. The cPLA10P4000-50 blend, with a T_m_ above 150 °C, exhibited both a high ε_b_ of 123% and a U of 107 kJ/m^2^.

The high drawability of amorphous PLA required good miscibility with the plasticizer, which led to a strong decrease in T_g_. However, the presence of a well-dispersed copolymer-rich phase with a low T_g_, dispersed within the amorphous PLA-rich phase in both the quenched and crystalline blends containing the P4000-50 copolymer—despite their relatively high T_g_ of 46 °C—was also beneficial for the mechanical performance. This shows that, in the partially miscible and phase-separated blends, both fractions of the copolymers—those molecularly dispersed, which plasticized the amorphous phase, and those phase-separated—contributed to the enhanced ability for plastic deformation, which improved the drawability and toughness. Furthermore, comparison with previously published results [52] shows that good dispersion of the copolymer-rich phase is favorable for the mechanical properties.

## Figures and Tables

**Table 1:** Thermogravimetric parameters of P2700-40 and P4000-50 copolymers, neat PLA, and its blends with the copolymers measured during heating at 20 °C/min in a nitrogen atmosphere and in air. T_5%_—temperature of 5% weight loss; T_d_—temperature of DTGA peak.

Sample Code	In Nitrogen		In Air	
	T_5%_ (°C)	T_d_ (°C)	T_5%_ (°C)	T_d_ (°C)
P2700-40	324	392	197	270
P4000-50	350	393	223	285
qPLA	334	382	331	367
qPLA5P2700-40	335	370	314	369
qPLA10P2700-40	325	369	295	372
qPLA5P4000-50	340	381	324	371
qPLA10P4000-50	322	379	306	375

**Table 2:** Calorimetric parameters of P2700-40 and P4000-50 copolymers based on DSC heating thermograms recorded at 10 °C/min. T_g_—glass transition temperature; T_cc_ and ΔH_cc_—cold crystallization temperature and enthalpy, respectively; T_m_ and ΔH_m_—melting peak temperature and enthalpy, respectively; X_c_—crystallinity degree of copolymer; X_cPEG_—crystallinity degree of PEG blocks.

Sample Code	T_g_ (°C)	T_cc_ (°C)	ΔH_cc_ (J/g)	T_m_ (°C)	ΔH_m_ (J/g)	X_c_ (%)	X_cPEG_ (%)
P2700-40	−68	−13	1.5	13	43	22	55
P4000-50	−72	-	-	26, 47	74	38	75

**Table 3:** Calorimetric parameters of quenched and crystallized PLA and its blends with P2700-40 and P4000-50 copolymers based on DSC heating thermograms recorded at 10 °C/min, and temperatures of loss modulus peaks determined by DMTA: T_g_—glass transitions temperature; T_cc_ and ΔH_cc_—cold crystallization temperature and enthalpy, respectively; T_m_ and ΔH_m_—melting peak temperature and enthalpy, respectively; X_cPLA_—crystallinity degree of PLA in materials; T_LE″_ and T_HE″_—temperatures of low- and high-temperature E’’ peaks. The bracket marks low-temperature shoulder of the high-temperature E” peak. ΔH_cc_ and ΔH_m_ of blends were recalculated per gram of PLA (g_PLA_).

Sample Code	T_g_ (°C)	T_cc_ (°C)	ΔH_cc_ (J/g_PLA_)	T_m_ (°C)	ΔH_m_ (J/g_PLA_)	X_cPLA_ (%)	T_LE″_ (°C)	T_HE″_ (°C)
qPLA	53	122	25.5	150	25.5	0	-	57
cPLA	55	99	2.0	151	35	31	-	58
qPLA5P2700-40	48	102	28.9	144, 153	30.3	1.3	-	45
qPLA10P2700-40	38	93	30.6	141, 152	31.6	0.9	(−20)	39
qPLA5P4000-50	46	120	21.4	155	22.4	0.9	−32	49
qPLA10P4000-50	46	113	29.4	153	29.6	0.2	−50	46
cPLA5P2700-40	43	-	-	145, 153	33.6	31.7	−38	52
cPLA10P2700-40	42	-	-	142, 153	34.8	33.0	−39	49
cPLA5P4000-50	46	94	10.8	151	35.0	22.8	−46	51
cPLA10P4000-50	46	-	-	151, 156	35.5	33.5	−64	51

**Table 4:** Mechanical parameters (averaged values) of quenched and crystallized PLA and its blends with P2700-40 and P4000-50 copolymers: E—Young’s modulus; σ_y_ and ε_y_—yield stress and strain, respectively; σ_b_ and ε_b_—stress and strain at break, respectively; U—tensile impact strength.

Sample Code	E (GPa)	σ_y_ (MPa)	ε_y_ (%)	σ_b_ (MPa)	ε_b_ (%)	U (kJ/m^2^)
qPLA	1.45	67	4.2	60	8.0	65
cPLA	1.55	-	-	68	5.0	34
qPLA5P2700-40	1.20	59	5.1	44	11.0	66
qPLA10P2700-40	0.87	46	7.5	38	560	70
qPLA5P4000-50	1.35	56	4.3	36	35	95
qPLA10P4000-50	1.30	44	3.4	26	240	90
cPLA5P2700-40	1.25	52	5.0	46	6.7	55
cPLA10P2700-40	0.93	35	5.0	26	36	120
cPLA5P4000-50	1.22	-	-	39	3.7	57
cPLA10P4000-50	1.25	34	3.0	27	123	107

**Table 5:** Characteristics of copolymers with different block structures supplied by BASF. Molar mass (M) and PEG content (P_c_) according to the information provided by the supplier.

Trade Name	Sample Code	M (g/mol)	P_c_ (wt%)
Pluronic^®^17R4	P2700-40	2700	40
Pluriol^®^WSB125	P4000-50	4000	50

## Data Availability

The data presented in this study are available on request from the corresponding authors.

## Abbreviations

The following abbreviations are used in this manuscript:

DMTA—dynamic mechanical thermal analysis; DSC—differential scanning calorimetry; DTGA—differential thermogravimetric analysis; SEC—size exclusion chromatography; SEM—scanning electron microscopy; TGA—thermogravimetric analysis.
